# C-856-08. The Impact of Dyschromic Burn Scar on Satisfaction with Appearance: A Burn Model System Study

**DOI:** 10.1093/jbcr/irag033.100

**Published:** 2026-04-06

**Authors:** Tharun Potluri, Bonnie C Carney, Ilona Argirion

**Affiliations:** The Burn Center at MedStar Washington Hospital Center, Washington, DC; The Burn Center at MedStar Washington Hospital Center, Washington, DC; Georgetown University School of Health, Washington, DC

## Abstract

**Introduction:**

Burn wounds can lead to hypertrophic scar formation, often accompanied by dyschromia, which has been shown to disproportionately affect patients with skin of color (SOC). Although the psychosocial impact of dyschromia is well-studied in diseases such as vitiligo and albinism, its impact on burn survivors remains unclear. As such, this study aimed to characterize the psychosocial impact of post-burn scar dyschromia.

**Methods:**

Data from 1352 subjects in the Burn Model System (BMS) National Database were analyzed. Study subjects self-assessed whether their burn scar was dyschromic at discharge from the hospital. In addition to demographics and injury characteristics, responses to a 14-item Satisfaction With Appearance (SWAP) scale were collected at discharge, 6, 12, and 24-months post-discharge. Associations were analyzed using bivariate and multivariable logistic regression.

**Results:**

In subjects who reported dyschromia at discharge (91.6%, n = 1239), the median age was 32 years, 68.2% (n = 845) were male, 44.6% (n = 553) had SOC, and the median total body surface area (TBSA) burned was 24.0%. Subjects with raised or thick scar (RTS) and female subjects had an 8-fold (OR = 8.61, CI = 5.28-14.00) and 2-fold (OR = 2.18, CI = 1.28-3.71) greater odds of reporting dyschromia, respectively. Those with SOC had higher odds of reporting resolved compared to persistent dyschromia across follow-up periods when controlling for RTS, age, and sex (OR = 1.42, CI = 1.35-1.97). When controlling for age, sex, SOC, etiology, burn and graft TBSA, and RTS, the association between dyschromia and dissatisfaction with appearance did not reach statistical significance (OR = 1.76, CI = 0.90-3.46). However, other predictors of decreased satisfaction were identified and included female sex (OR = 1.69, CI = 1.11-2.57), SOC (OR = 2.84, CI = 1.84-4.39), >60% TBSA burn (OR = 7.71, CI = 1.65-36.02), 21-40% TBSA requiring graft (OR = 2.35, CI = 1.12-4.94), and RTS (OR = 2.79, CI = 1.84-4.23).

**Conclusions:**

While dyschromia did not have a statistically significant impact on satisfaction with appearance in burn survivors, subjects who had SOC and who reported RTS, both of which are associated with dyschromia, had higher odds of dissatisfaction. Further studies, combined with the use of increasingly robust and objective measures of dyschromia, should be conducted to better understand the psychosocial impact of dyschromia on burn survivors.

**Applicability of Research to Practice:**

Dyschromia remains an under-recognized consequence of burn injury. Characterizing the impact of dyschromia on the psychosocial recovery of burn patients may help guide attention towards the importance of identifying and implementing effective treatments that could improve quality of life for burn survivors.

**Funding for the study:**

This work was funded in part by an award from the National Center for Advancing Translational Science (NCATS/NIH).^1^

